# Genetic evidence for the rediscovery in the wild of the critically endangered Sahara killifish *Apricaphanius saourensis* (Cyprinodontiformes: Aphaniidae)

**DOI:** 10.1007/s00114-026-02074-7

**Published:** 2026-01-29

**Authors:** Louiza Derouiche, Redouane Tahri, Carlos Rodríguez Fernandes

**Affiliations:** 1 ESSAIA - Higher School of Food Sciences & Agri-food Industries, 16200 El Harrach, Algiers, Algeria; 2 Wild Algeria, Béchar, Algeria; 3 CE3C - Centre for Ecology, Evolution and Environmental Changes & CHANGE - Global Change and Sustainability Institute, Departamento de Biologia, Faculdade de Ciências, Universidade de Lisboa, Lisboa, 1749-016 Portugal; 4 Faculdade de Psicologia, Universidade de Lisboa, Alameda da Universidade, Lisboa, 1649-013 Portugal

**Keywords:** Aphaniid, Maghreb, Northwest Africa, Genetic species identification, Mitochondrial DNA, Nuclear DNA

## Abstract

*Apricaphanius saourensis* was described in 2006 from the Saoura River in western Algeria, and is currently listed as possibly extinct in the wild. We recently discovered an aphaniid population in a very isolated secondary wadi of the Guir River about 115 Km northwest of *A*. *saourensis’* type locality, which we hypothesized could belong to *A*. *saourensis* based on images taken from living individuals. We report here results of mitochondrial and nuclear DNA analyses that suggest that this Guir River population indeed represents the rediscovery in the wild of *A*. *saourensis*.

## Introduction

**Fig. 1:**
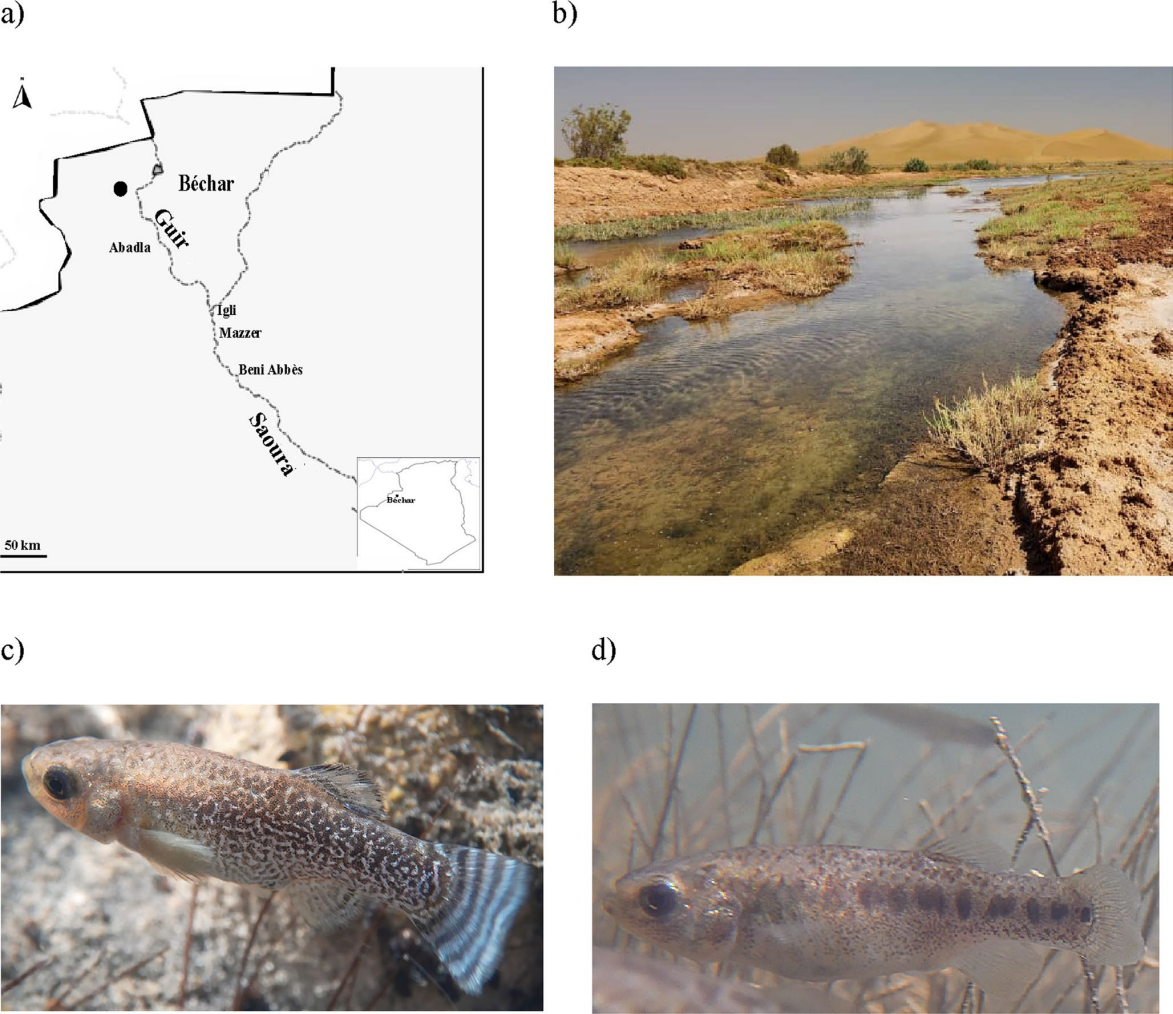
(**a**) Map showing the location of the wadi (indicated by a black dot) in the Guir River valley in the Abadla district, Béchar Province. Also indicated are the type locality of *A*. *saourensis* (Mazzer) and neighbouring main towns in the Saoura River valley (Igli and Béni Abbès). The grey dotted lines are the main rivers in the region, and the grey triangle on the Guir River represents the Djorf Torba dam. The thick black line outlines the Algerian border with Morocco, to the northwest of the map. The inset map on the bottom right shows the location of the Béchar region within Algeria; (**b**) Photograph of the wadi in the Guir River valley where we found the aphaniid population analysed here; (**c**) Example of a male individual from that population; (**d**) Example of a female individual from the same population

The Sahara killifish *Apricaphanius saourensis *(Blanco, Hrbek & Doadrio, 2006) (Blanco et al. 2006) is a cyprinidontiform freshwater fish of the Aphaniidae family that was described from the Saoura River in Mazzer, western Algeria. The species was originally assigned to the genus *Aphanius* (Blanco et al. 2006), but has been reclassified in *Apricaphanius* (Freyhof and Yoğurtçuoğlu 2020). Blanco et al. (2006) showed that the species is distinct, both morphologically and genetically, from the other two known congeneric species: *A. iberus* (Valenciennes, 1846) (Cuvier and Valenciennes 1846) (Spanish killifish) and *A. baeticus* (Doadrio, Carmona & Fernández-Delgado, 2002) (Doadrio et al. 2002) (Baetican killifish). Based on data for the complete mitochondrial cytochrome b (*Cyt b*) gene, these authors estimated a divergence between *A. saourensis* and *A. iberus* of 4.9–5.8% and between *A. saourensis* and *A. baeticus* of 6.3–6.5%. *Apricaphanius saourensis* still exists in captivity, in collections founded by individuals collected by Blanco et al. (2006) and transferred to European institutions, but it has not been observed in the wild since the time of its description, despite efforts to find it (Bacha et al. 2014; Freyhof and Ford 2022). The apparent extinction of *A. saourensis* in the area where it was discovered is generally considered to be the result of a potential combination of several factors detrimental to the species: the presence and expansion of invasive species (Kara 2012), such as the eastern mosquitofish *Gambusia holbrooki* Girard 1859 and the Nile tilapia *Oreochromis niloticus* (L. 1758), which compete with aphaniids; water consumption from the river for human use; water pollution; and increasingly frequent extreme drought events (Freyhof and Ford 2022). Given the absence of records of *A. saourensis* in the wild since its description, the current conservation status of the species is critically endangered and possibly extinct in the wild (Freyhof and Ford 2022), with prospecting for the species in the Saoura river valley and neighbouring wadis being considered a priority (Freyhof and Ford 2022). One of us (Redouane Tahri) conducted such surveys for several years until, in late June 2024, he found an aphaniid population that may belong to *A*. *saourensis* in a very isolated secondary wadi (31° 12’ 7.89” N 2° 53’ 42.93” W) of the Guir River, which flows into the Saoura (Tahri et al. 2025). The wadi is located in the Abadla district, at approximately 115 km northwest of *A*. *saourensis*’ type locality (Fig. 1a). The fact that the wadi is far from human settlement and concomitant anthropogenic pressure, such as water withdrawal for agricultural use and pollution, likely contributed to the persistence of the aphaniid population (Fig. 1b). Comparisons of images of living individuals from said population with characteristics of the body pigmentation patterns mentioned as diagnostic for *A*. *saourensis* (Blanco et al. 2006) allowed the identification of colour morphotypes in both sexes that resemble *A*. *saourensis*, but also of phenotypes distinct from those presented in Blanco et al. (2006). Specifically, those distinct phenotypes observed in the Guir River wadi are characterized by a much less conspicuous blue mottling of the body in males compared to the male specimen of *A*. *saourensis* presented in Blanco et al. (2006; Fig. 4), and the presence of a row of large black spots along the lateral line in the posterior half of the trunk in females, something completely absent in the female specimen of *A*. *saourensis* presented in Blanco et al. (2006; Fig. 5) (Tahri et al. 2025). The distinct phenotypes in the Guir River are also clearly different from the known morphotypes in the extant congeneric species *A*. *baeticus* and *A*. *iberus* (Blanco et al. 2006; Freyhof and Yoğurtçuoğlu 2020). Figure 1c and d show photographs of a male and a female individual from the Guir River population, respectively. It is however important to note that interpopulation morphological differentiation, including in body pigmentation patterns, has been observed within other aphaniid species (Bidaye et al. 2023; Teimori et al. 2018, 2021). Genetic analysis can be extremely useful and powerful in efficiently clarifying such uncertainties in taxonomic identification (e.g. Pereira et al. 2013; Melo et al. 2014; Jense et al. 2024), and has already proven instrumental in the rediscovery of other freshwater fish species previously thought to be possibly extinct and in understanding their current distribution (Vukić et al. 2016, 2017; Wibowo et al. 2023; Mokodongan et al. 2025). Here we present mitochondrial and nuclear DNA analyses of specimens from the recently discovered aphaniid population in the Guir River of uncertain species identity, and comparison with homologous DNA sequence data for the type population of *A*. *saourensis* and for the other two known *Apricaphanius* species. The results suggest that the study population belongs to *A. saourensis*.

## Materials and methods

Following the discovery of the aphaniid population, we made underwater recordings and captured images of live individuals to compare with *A*. *saourensis*, and reported the observations (Tahri et al. 2025). Subsequently, we decided to return to the wadi and tried to find and collect dead individuals, in recently dried pools or about to dry completely, for genetic analysis. We collected 20 specimens, which were placed in tubes filled with absolute ethanol and kept refrigerated until arrival at the laboratory, where they were stored in a freezer at −20 °C until analysis. Genomic DNA was extracted from muscle samples using the EZNA Tissue DNA kit (Omega Bio-Tek). For polymerase chain reaction (PCR) amplification and Sanger sequencing of the entire *Cyt b* gene, we designed two primer pairs based on an alignment of mitochondrial DNA sequences available in GenBank (https://www.ncbi.nlm.nih.gov/genbank/) for the three *Apricaphanius* species. The primer pairs are Asaourensis Cytb F1 (5’-CCAGGACTAATGGCTTGA-3’) and Asaourensis Cytb R1 (5’-TTATTTGAGCCTGATTCG-3’), and Asaourensis Cytb F2 (5’-AGGGGGCTTCTCAGTTGA-3’) and Asaourensis Cytb R2 (5’-TTTAACCCTCGACATCCG-3’). Similarly, we designed a pair of primers, based on an aphaniid alignment, to amplify and sequence 535 bp of the nuclear gene *RAG1*: Asaourensis RAG1 F1 (5’-GGCTTTAGAGTCATGATC-3’) and Asaourensis RAG1 R1 (5’-CTTTGACTCTGTCTCGCA-3’). The choice of this gene and the specific gene fragment selected for the nuclear DNA analysis was due to the existence of a published homologous sequence of *A*. *saourensis* from Mazzer. For the three primer pairs, PCRs were carried out in volumes of 15 µl with 0.3 µM of each primer, 1.5 U of Multiplex PCR NZYTaq 2x Colourless Master Mix (NZYTech), and 4 µl of DNA extract. Thermal cycling conditions consisted of an initial denaturation at 95 °C for 5 min, followed by 45 cycles of 30 s at 94 °C, 30 s at 55 °C, 30 s at 72 °C, and a final extension of 7 min at 72 °C. The results of the PCR amplifications were visualized on 2% agarose gels to verify PCR quality, and the PCR products were purified with an Exo-SAP protocol (Hanke and Wink 1994; Werle et al. 1994) and sequenced at Macrogen Inc.

Sequences were edited, assembled, aligned, and checked for the absence of indels and stop codons using Sequencher 4.7 (Gene Codes Corporation). We downloaded *Cyt b* sequences among the longest available in GenBank for the three *Apricaphanius* species (for *A*. *saourensis* only one *Cyt b* sequence was available, published by Blanco et al. (2006)), and added them to our sequence dataset (Table 1). Specifically, we downloaded 20 sequences from each of the two congeneric species of *A*. *saourensis* (*A*. *iberu*s and *A*. *baeticus*), in both cases from across their geographic range (Perdices et al. 2001; Gonzalez et al. 2014, 2018), to obtain a good representation of the mitochondrial genetic diversity in each species. We also downloaded the *RAG1* sequence of *A*. *saourensis* available from GenBank (accession number KJ844662; Pohl et al. 2015). Sequence alignments were analysed with FaBox 1.61 (https://users-birc.au.dk/~palle/php/fabox/index.php; Villesen 2007) to collapse sequences into haplotypes or alleles. File format conversions of sequence alignments for use in different computer programs were done using ALTER (https://www.sing-group.org/ALTER/; Glez-Peña et al. 2010). Estimates of gene and nucleotide diversity were obtained using Arlequin 3.5.2.2 (Excoffier and Lischer 2010). To visualize the genealogical relationships among *Cyt b* haplotypes or *RAG1* alleles, we built haplotype networks in PopART 1.7 (Leigh and Bryant 2015). We also inferred a phylogenetic tree for the *Apricaphanius Cyt b* sequences with the Bayesian method implemented in MrBayes 3.2.6 (Ronquist et al. 2012). Based on the results of Esmaeili et al. (2020), which suggest a close phylogenetic relationship between the genera *Apricaphanius* and *Esmaeilius*, we used a sequence from the Zagros toothcarp *Esmaeilius vladykovi* (Coad 1988) (Genbank accession number DQ367526) as an outgroup for the *Apricaphanius* phylogenetic tree. Analyses in MrBayes were conducted with two parallel Markov Chain Monte Carlo (MCMC) runs, each with four Markov chains (one cold and three heated), default heating parameter (*t* = 0.1), and 20 million generations. The first five million generations were discarded as burn-in and, thereafter, chains were sampled every 500 generations. The entire general time-reversible (GTR; Lanave et al. 1984) substitution model space was sampled within the analyses (Huelsenbeck et al. 2004), and the sequence alignment was partitioned by codon position, as this was the best-fit partitioning scheme according to the corrected Akaike information criterion (AICc) (Akaike 1974) in PartitionFinder 2.1.1 (Lanfear et al. 2017) using PhyML (Guindon et al. 2010). Convergence was indicated by an average standard deviation of split frequencies between parallel runs of less than 0.01. For all model parameters, the minimum and average effective sample sizes (ESS) among runs were greater than 700 and 4850, respectively, and the potential scale reduction factor (PSRF) was 1.0. Convergence to the stationary distribution for model parameters on each of the runs was also confirmed by analysis of the MCMC samples in Tracer 1.7.2 (Rambaut et al. 2018). Support for tree nodes was determined according to the values of Bayesian posterior probability (BPP) obtained in a majority-rule consensus tree (Berry and Gascuel 1996; Holder et al. 2008; Huggins et al. 2011). The majority-rule consensus tree was computed with SumTrees 4.5.2 of the DendroPy library version 4.5.2 (Sukumaran and Holder 2010) and visualized and edited with FigTree 1.4.4 (available at https://github.com/rambaut/figtree).

**Table:** Information on the analysed *Cyt b* sequences

Population/species	Country	Sequence	Haplotype	Reference
Newly discovered aphaniid population (Abadla)	Algeria	PV932924 PV932925	S1 S2	This study This study
*A*. *saourensis type population (Mazzer)*	Algeria	DQ367527	S3	Blanco et al. 2006
*A*. *iberus*	Spain	AF299274	I1	Perdices et al. 2001
	Spain	AF299275	I2	Perdices et al. 2001
	Spain	AF299276, AF299277	I3	Perdices et al. 2001
	Spain	AF299278	I4	Perdices et al. 2001
	Spain	AF299279	I5	Perdices et al. 2001
	Spain	AF299286	I6	Perdices et al. 2001
	Spain	AF299287	I7	Perdices et al. 2001
	Spain	AF299288	I8	Perdices et al. 2001
	Spain	AF299289	I9	Perdices et al. 2001
	Spain	AF299290	I10	Perdices et al. 2001
	Spain	AY155569	I11	Doadrio and Dominguez 2004
	Spain	DQ367528	I12	Blanco et al. 2006
	Spain	DQ367529	I13	Blanco et al. 2006
	Spain	KU174219	I14	Gonzalez et al. 2018
	Spain	KU174223	I15	Gonzalez et al. 2018
	Spain	KU174226	I16	Gonzalez et al. 2018
	Spain	KU174230	I17	Gonzalez et al. 2018
	Spain	KU174231	I18	Gonzalez et al. 2018
	Spain	NC072677	I19	López-Solano et al. 2023
*A*. *baeticus*	Spain	KF854343	B1	Gonzalez et al. 2014
	Spain	KF854345	B2	Gonzalez et al. 2014
	Spain	KF854395, KF854416	B3	Gonzalez et al. 2014
	Spain	KF854402	B4	Gonzalez et al. 2014
	Spain	KF854411	B5	Gonzalez et al. 2014
	Spain	KF854419, KF854420, KF854426, KF854427, KF854428	B6	Gonzalez et al. 2014
	Spain	KF854395, KF854416	B7	Gonzalez et al. 2014
	Spain	KF854442, KF854443, KF854449, KF854453, KF854454	B8	Gonzalez et al. 2014
	Spain	KF854476	B9	Gonzalez et al. 2014
	Spain	KF854477	B10	Gonzalez et al. 2014
*E*. *vladykovi*	Iran	DQ367526	E_vladykovi	Blanco et al. 2006

## Results and discussion

**Fig. 2:**
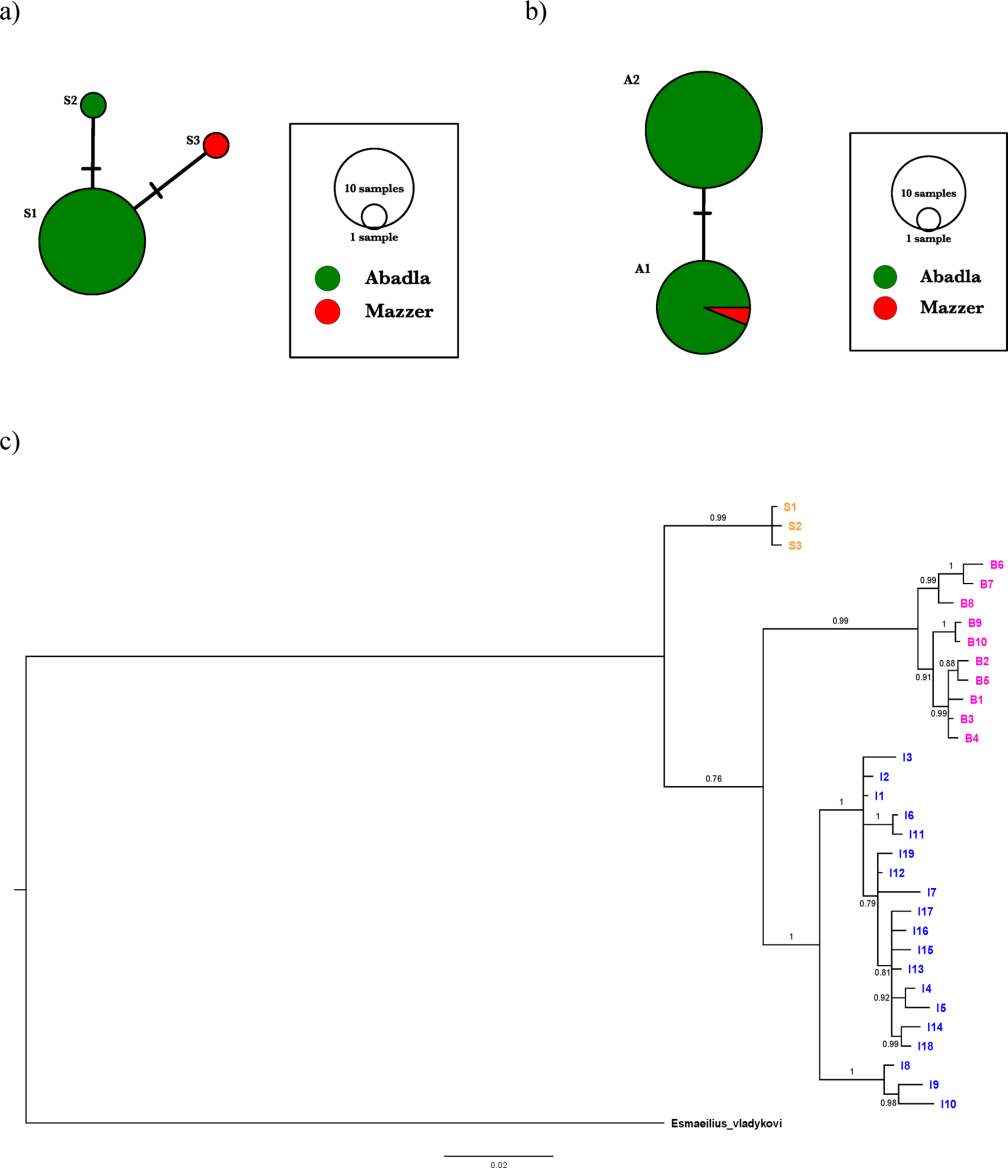
(**a**) Network of known *A*. *saourensis Cyt b* haplotypes. The circles represent haplotypes, with their size proportional to their frequency and coloured according to where they were found. Dashes on lines connecting haplotypes represent the number of nucleotide substitutions separating them. Haplotype designations and further information are given in Table 1; (**b**) Network of *A*. *saourensis RAG1* alleles. The circles represent alleles, with their size proportional to their frequency and coloured according to where they were found. Dashes on lines connecting alleles represent the number of nucleotide substitutions separating them; (**c**) Majority-rule consensus phylogram (cut-off 0.7) from the Bayesian inference analysis of the *Apricaphanus Cyt b* haplotypes dataset rooted with *Esmaeilius vladykovi*. Haplotypes are named and coloured according to species identity (initial ‘S’ and orange for *saourensis*, initial ‘B’ and magenta for *baeticus*, and initial ‘I’ and blue for *iberus*). See Table 1 for haplotype information. Numbers indicate node support in terms of Bayesian posterior probabilities. The scale at the bottom is in units of nucleotide substitution per site

We obtained complete *Cyt b* sequences of 1140 base pairs (bp) for 19 of the 20 samples analysed in the laboratory (for sample 12 we obtained the first 1113 bp of the gene). The 19 sequences corresponded to two haplotypes, which we designated S1 and S2, with the latter present only in sample 2 (for the 1113 bp of sample 12 the sequence was identical to that of haplotype S1). These S1 and S2 haplotypes were different from the only known *Cyt b* sequence of *A*. *saourensis* (from the Saoura River in Mazzer, Genbank accession number DQ367527, which we here designate as haplotype S3), and therefore we deposited their sequences in GenBank (accession numbers PV932924-PV932925). Based on the 1113 bp dataset of 20 sequences, the estimates and standard deviations of haplotype and nucleotide diversity for the population in the Guir River wadi were 0.100 ± 0.088 and 0.00009 ± 0.00018, respectively. The genealogical relationships between haplotypes S1-S3 are depicted in the haplotype network in Fig. 2a, with the genetic proximity between the three haplotypes being evident, with genetic distances of only one to two mutational steps between the haplotypes. The close proximity between the Guir River haplotypes and the Mazzer haplotype is also evidenced in a phylogenetic tree for *Apricaphanius* (Fig. 2c), based on an alignment of 982 bp (positions 132–1113 of the *Cyt b* gene). The topology of this tree is similar to that inferred for the genus by Esmaeili et al. (2020) using the mitochondrial cytochrome oxidase I gene (COI).

For the *RAG1* fragment, we obtained a 418 bp alignment for the 20 analysed samples from the Guir River, with forward and reverse sequences for each sample. We found two *RAG1* alleles, differing by a single nucleotide substitution, in the population sample. The observed heterozygosity was 0.550 (i.e., 11 individuals were heterozygous and 9 individuals were homozygous). The estimated gene and nucleotide diversity were 0.481 ± 0.042 and 0.00115 ± 0.00113, respectively. To compare the two alleles found with the 433 bp *RAG1* sequence from *A*. *saourensis* downloaded from GenBank, we generated a fully overlapping alignment between this sequence and the 418 bp dataset. This alignment, with 358 bp, was used to estimate the network shown in Fig. 2b. As can be observed, one of the two alleles we found in the Guir River wadi is the same as the one previously reported for *A*. *saourensis* from Mazzer. We deposited a sequence containing the other allele in GenBank (accession number PX781542). For the sake of simplicity, we designated the former allele as A1 and the latter as A2. The observed frequency of allele A1 in the Guir River population sample was 0.375, and therefore the observed frequency of allele A2 was 0.625.

In light of the results obtained from the *Cyt b* and *RAG1* analyses for the individuals sampled from the Guir River wadi, with DNA sequences identical or very similar to those observed in the type population of *A*. *saourensis*, the most parsimonious interpretation seems to be to consider the Guir River population as also belonging to this species. It is true that this conclusion is based on patterns observed for only one nuclear gene and one mitochondrial gene, and that the divergence between the Guir River and Mazzer lineages should be evaluated in the future with nuclear genome-wide data (e.g. Pedraza-Marrón et al. 2019; Campbell et al. 2022). But for now we believe that the most supported working hypothesis is to consider that the Guir River aphaniid represents the rediscovery of *A*. *saourensis* in the wild.

Resolving this taxonomic issue is important, but equally crucial is ensuring the protection and conservation of the recently discovered population in the Guir River wadi. Given the various efforts to find extant populations of *A*. *saourensis* across the provinces of Béchar and Béni Abbès (Bacha et al. 2014; Tahri et al. 2025), which proved fruitless with the exception of the Guir River population analysed here, it is feasible that this may be the last remaining wild population of *A*. *saourensis*. In any case, given that this study indicates that *A*. *saourensis* is present in a location reasonably distant from the type locality, we believe that the search for the species should continue in other locations in the region that have not yet been explored.

Our results suggest low genetic variation in the Guir River population. A priority for future work will be to verify this suggestion with analysis of extensive genome-wide nuclear DNA datasets. Low genome-wide genetic diversity tends to be negatively associated with the ability to persist in the face of environmental adversities (which characterize the ecosystem in which the Guir River population lives) and for adaptive evolution (DeWoody et al. 2021; Kardos et al. 2021). It is true that the included haplotypes of *A*. *baeticus* and *A*. *iberus* (Table 1) correspond to individuals from several populations of each species, but the combined *Cyt b* results for Guir River and Mazzer suggest that genetic diversity may be much lower in *A*. *saourensis*. For example, for comparison with the estimates obtained here for the Guir River population, Gonzalez et al. (2014) found 58 haplotypes in 137 *A*. *baeticus* individuals analysed for 1009 bp of the *Cyt b* gene, and estimated a haplotype and nucleotide diversity of 0.895 ± 0.014 and 0.010 ± 0.005, respectively. Similarly, for *A*. *iberus*, Gonzalez et al. (2018) found 59 haplotypes in 173 individuals analysed for 1117 bp of the *Cyt b* gene, and estimated a haplotype and nucleotide diversity of 0.850 ± 0.030 and 0.005 ± 0.001, respectively. The significantly lower mitochondrial genetic diversity in *A*. *saourensis* compared to the two congeneric species may be due to the fact that the former has undergone a much more intensive recent history of population extinctions and drastic demographic decline (e.g. Hurt et al. 2017; Jang et al. 2017). However, considering the numerous examples of species that have suffered significant recent demographic contractions and still exhibit substantial mitochondrial genetic variation (Liu et al. 2012; Souza-Shibatta et al. 2018; Radhakrishnan et al. 2026), it is feasible that the very low mitochondrial genetic diversity observed in *A*. *saourensis* could also result from a history with recurrent severe demographic contractions, as a consequence of episodes of extreme aridity that are frequent in the distribution range of the species (Wengler and Vernet 1992; Zielhofer et al. 2008; Yahiaoui et al. 2023). Indeed, *A*. *saourensis* lives in an area where climatic conditions are conducive to regular events of extreme drought in which water bodies disappear or shrink massively, and which will likely correspond to periods of significantly reduced population size. Repeated demographic bottlenecks are expected to accelerate the loss of genetic diversity through increased genetic drift and inbreeding and reduced gene flow (Vucetich et al. 1997; Montgomery et al. 2000; Landergott et al. 2001; Parra et al. 2018).

It is vital that protection and support be offered to the population in the Guir River wadi so that it can survive in the long term. In this context, it is a cause for hope and rejoicing that the Algerian government has decided to provide legal protection for the wadi area and the aphaniid population living within it, and has established a captive breeding program for the population (Tahri et al. 2025).

## Data Availability

The datasets analysed during this study are available from the corresponding author on reasonable request.
